# On Emergence of Spontaneous Oscillations in Kombucha and Proteinoids

**DOI:** 10.1007/s12668-024-01678-5

**Published:** 2024-12-05

**Authors:** Panagiotis Mougkogiannis, Anna Nikolaidou, Andrew Adamatzky

**Affiliations:** Unconventional Computing Laboratory, University of the West of England, Bristol, BS16 1QY UK

**Keywords:** Kombucha, Proteinoids, Spontaneous oscillations, Chaos, Self-organisation, Origin of life

## Abstract

An important part of studying living systems is figuring out the complicated steps that lead to order from chaos. Spontaneous oscillations are a key part of self-organisation in many biological and chemical networks, including kombucha and proteinoids. This study examines the spontaneous oscillations in kombucha and proteinoids, specifically exploring their potential connection to the origin of life. As a community of bacteria and yeast work together, kombucha shows remarkable spontaneous oscillations in its biochemical parts. This system can keep a dynamic balance and organise itself thanks to metabolic processes and complex chemical reactions. Similarly, proteinoids, which may have been primitive forms of proteins, undergo spontaneous fluctuations in their structure and function periodically. Because these oscillations happen on their own, they may play a very important part in the development of early life forms. This paper highlights the fundamental principles governing the transition from chaos to order in living systems by examining the key factors that influence the frequency and characteristics of spontaneous oscillations in kombucha and proteinoids. Looking into these rhythms not only helps us understand where life came from but also shows us ways to make self-organising networks in synthetic biology and biotechnology. There is significant discussion over the emergence of biological order from chemical disorder. This article contributes to the ongoing discussion by examining at the theoretical basis, experimental proof, and implications of spontaneous oscillations. The results make it clear that random oscillations are an important part of the change from nonliving to living matter. They also give us important information about what life is all about.

## The Emergence of Order: Spontaneous Oscillations

Spontaneous electrical low-frequency oscillations (SELFOs) have been detected in several living systems, ranging from simple organisms like Hydra to complex structures like the human brain; see overview in [1]. These oscillations, which usually occur between 0.01 and 0.1 Hz, seem to be produced without being influenced by external factors and do not directly play a role in behaviour generation. The existence of SELFOs in several phyla implies that they may fulfil a fundamental biological role that originated in the early stages of life on Earth.

Recent research has indicated that SELFOs are not exclusive to organisms possessing neural systems. Spontaneous electrical activities have been observed in nonneuronal organisms, including plants [2, 3], fungus [4–6], slime mould [7], protozoa [8], bacteria [9], and moss [10]. The prevalence of SELFOs throughout the living world prompts fascinating inquiries on their contribution to the structure and operation of living systems.

This research examines the notion of SELFOs as electrical organism organisers. SELFOs play a crucial role in integrating and communicating within the entire system, which is essential for establishing and sustaining unity and coherent, adaptive behaviour in organisms. Our primary areas of interest are kombucha and proteinoids, which are both interesting chemical systems. Both kombucha, a combination of bacteria and yeast, and proteinoids, synthetic polypeptides that closely resemble natural proteins, have demonstrated spontaneous oscillations, as documented in the study by Mougkogiannis et al. [11].

Through the analysis of the spontaneous oscillations in these apparently unrelated systems, our objective is to provide insight into the underlying principles that regulate the shift from disorder to organisation in living systems [12].

Our hypothesis suggests that SELFOs found in kombucha and proteinoids may contribute to the self–organisation and coordination of these complex systems, similar to the suggested role of SELFOs in other living organisms.

In addition, we investigate the possible consequences of SELFOs in kombucha and proteinoids for gaining insights into the beginning of life and advancing the field of living electronics. The existence of self-generated oscillations in these systems indicates that they could be useful models for investigating the development of biological organisation from chemical disorder [13].

In our study, we seek to expand the existing research on the role of bioelectricity as a “template” for developmental memory in organism regeneration. We achieve this by comparing SELFOs in kombucha, proteinoids, and other living systems. Investigating the spontaneous oscillations in these various systems can offer useful insights into the underlying principles that control the organisation and operation of life systems, ranging from the simplest organisations to the most complex ones [1].

### Spontaneous Neural Oscillations in the Human Brain

The discovery of spontaneous, rhythmic electrical activity in the human brain dates back to 1929, when Hans Berger invented the electroencephalogram (EEG) and identified what he termed “alpha waves” [14]. Despite this groundbreaking finding, the prevailing view of the brain as an input–output machine, only active in response to external stimuli, dominated neuroscience for nearly seven decades [15]. It was not until the late 1990s that neuroscientists Shulman and Raichle independently observed a paradoxical result in human neuroimaging studies designed to detect task-evoked activity. They found a specific brain network that appeared to be inhibited during tasks and more active when subjects were at rest with their eyes closed [16, 17].

This discovery questioned the traditional notion that only external stimuli can generate brain activity. Later research confirmed that intrinsic brain activity can happen even when there are not any changes in the environment or goal-directed behaviour. This led Shulman and Raichle to believe that the traditional view was very wrong. The spontaneous, resting-state network they identified became known as the brain’s “default mode network” (DMN) and quickly became a focal point of neuroscientific research [16].

The identification of the default mode network (DMN) and its inherent neuronal oscillations in humans has significantly transformed our understanding of brain functionality. Recent findings have shown that the human brain is not simply a passive machine that responds to stimuli. Instead, it is a dynamic and self-organising system that produces complex patterns of activity even without any external input. The inherent brain activity, which utilises a substantial amount of the brain’s energy resources [18], is currently acknowledged as a key element of brain function and has been associated with many cognitive processes, such as self-referential thinking, wandering of the mind, and consolidation of memory [19].

The spontaneous neural oscillations observed in the human brain span a wide range of frequencies, from ultraslow (0.01–1.0 Hz) to ultrafast (200–600 Hz) [20, 21]. Mammalian species highly conserve this frequency distribution, indicating its critical role in brain function [21]. The DMN, in particular, has been found to oscillate in the ultraslow frequency range (0.01–0.1 Hz) [22, 23], which, combined with its central position in the brain’s network architecture, has led to the hypothesis that it may serve as the brain’s ultimate information integrator [24].

### Spontaneous Electrical Low-Frequency Oscillations (SELFOs) in the Living World

Throughout the natural world, a wide range of organisms, not just humans, exhibit SELFOs, or self-organising systems. In 1964, researchers conducted electrophysiological studies on two types of freshwater amoebae, *Chaos chaos* and *Amoeba proteus*, which resulted in unexpected findings [8]. During the analysis of high potassium levels in the cytoplasm of these single-celled eukaryotes, scientists discovered low-frequency “spike potentials” that exhibited similar behaviour to action potentials. Several substances, including ethyl ether, cocaine, potassium oxalate, and CaCl_2_, were shown to influence the spontaneous “spike potentials”. However, these substances did not have any noticeable impact on the behaviour or structure of the cell, making their function uncertain [8].

Until the development of a fluorescent voltage-sensitive protein in 2011, very little was known about the electrophysiology of bacteria in the world of prokaryotes. Researchers were able to see the dynamic electrical characteristics of bacteria, which showed spontaneous, low-frequency spikes similar to action potentials in *Escherichia coli*. These spikes were not directly linked to the bacteria’s behaviour [9]. Moreover, studies have demonstrated that *Bacillus subtilis* in biofilms may communicate across vast distances using synchronised low-frequency potassium waves, which coordinate the distribution of nutrients both inside and between biofilms [12, 25]. This discovery implies that low-frequency electrical oscillations may play a role in the overall integration and transfer of information throughout an organism.

Despite the lack of a clear understanding of their function, the majority of investigated organisms contain SELFOs, indicating their potential significant role in biological systems. These oscillations can be found in a wide range of organisms, from simple single-celled eukaryotes and prokaryotes to complex multicellular ones. This suggests that they may be a natural part of biological systems. Additional investigation is required to clarify the precise roles and mechanisms of SELFOs in different organisms and fully understand their evolutionary importance in the natural environment.

### The Emergence of SELFOs in Biological Systems: From Subcellular to Organismal Levels

In order to understand the emergence of SELFOs in biological systems, it is crucial to examine the collective behaviour of inanimate systems, where individual subunits at a smaller scale can generate emergent characteristics at a larger scale. Within nonliving systems, three fundamental emergent phenomena might arise: complete order, complete disorder, or a state that is somewhere in between [26]. Biological systems are complex and dynamic systems that have a tendency to organise themselves and stay in a state known as the “edge of chaos”. This state allows for maximum flexibility, enabling better flow of information and adaptation within the system [27, 28].

An unresolved question in the field of biology pertains to the mechanisms by which biological systems sustain their functionality within a highly constrained range of operation. The transmission of feedback from higher levels of scale to lower-level subunits is considered to be of utmost importance [29–31]. SELFOs can offer electrical top-down feedback to biological systems, helping to sustain them within a dynamic and functional state space.

Recent research has questioned the traditional belief that neuronal cells possess exclusive capabilities for transmitting electrical information. Several types of cells, including nonneural cells such as bacteria and numerous human cells, display electrical activity characterised by subthreshold membrane potential oscillations and action potentials similar to those observed in neurons [32–34]. These processes are commonly believed to be caused by the movement of ions through ion channels in cell membranes. Recent research indicates that proteins, contrary to previous belief, can carry substantial electrical current based on their structure [35]. This discovery implies that proteins can function as subcellular electrical components, resembling electronic elements. Different alterations can activate or deactivate these proteins, thereby impacting their capacity to carry electrical current.

Albert Szent-Györgyi first proposed this novel viewpoint in 1941 [36], suggesting that proteins with remarkably orderly configurations could act as electron semiconductors within cells, similar to inorganic substances like crystals. Michael Berry’s “electrochemical model of metabolism” [37, 38] posits that cellular metabolic pathways can only be fully understood by considering both chemical and electrical flows. According to this model, the movement of electrons and protons through proteins plays a crucial role in facilitating chemical reactions across the entire cell.

Mounting evidence indicates that in multicellular organisms lacking nervous systems, somatic cells engage in electrical communication through two mechanisms: direct communication, which involves ion flow through cell–cell gap junctions, and indirect communication, which involves extracellular ion flow through ion channels [34, 39]. The body-wide subthreshold membrane potential circuits play an essential role in both the development and maintenance of the overall body structure. They have been suggested as a possible “top-down” mechanism that organisms employ to coordinate their many parts [39, 40].

In organisms that have nervous systems, SELFOs can be produced through a “hierarchy of pacemakers,” as first suggested by Passano for the neural system of cnidarians [41, 42]. The majority of neurons demonstrate inherent pacemaker activity [43, 44], and when interconnected, they can spontaneously organise into ensembles, which are groupings of oscillators that oscillate at the same frequency. Thus, various types of neurological systems have the ability to autonomously organise themselves into more complex structures, creating a “hierarchy of pacemakers” where the largest and slowest oscillator in the system can regulate and limit the activities of the smaller and faster oscillators.

Overall, the presence of SELFOs in biological systems seems to be an essential characteristic that results from complex interactions occurring at several levels of organisation, ranging from subcellular protein networks to multicellular somatic cell circuits and neuronal ensembles. These oscillations can function as a hierarchical mechanism to organise and limit the lower-level elements, keeping the system in a dynamic and liveable condition and allowing for increased information transmission and flexibility.

Table 1 provides a summary of the parallels and differences among spiking oscillations in diverse biological and living systems. Electrical oscillations seem to be an inherent characteristic of complex systems, ranging from proteins to neural organisms. Proteins may function as subcellular electrical “hardware”, contributing to extensive intracellular electrical networks, by means of their electrical conductivity. Nonneural cells engage in intercellular electrical communication by exhibiting subthreshold membrane potential oscillations and action potentials resembling those of neurons. Nonneural organisms demonstrate SELFOs, which could potentially function as a mechanism for top-down coordination. Extracellular ion transport and gap junctions facilitate electrical communication. SELFOs, which may have originated from a “hierarchy of pacemakers” and function as a means to synchronise and restrict lower-level components, manifest an extremely conserved oscillation frequency structure in neural organisms.

**Table 1:** Comparison of spiking oscillations in living and biological systems

System	Similarities	Differences
**Proteins**	Exhibit electrical conductivity May act as subcellular electrical “hardware” part of complex intracellular electrical networks	Conductivity depends on protein conformation Modifications affect current flow
**Nonneural cells**	Exhibit subthreshold membrane potential oscillations. Display neuron-like action potentials Participate in intercellular electrical communication	Oscillations arise from ion flow through membrane channels Specific oscillation patterns may vary between cell types
**Nonneural organisms**	Exhibit spontaneous electrical low-frequency oscillations (SELFOs)	Oscillations may be generated by different mechanisms
	SELFOs may serve as a top-down coordination mechanism	Specific functions of SELFOs may vary between organisms
	Electrical communication occurs via gap junctions and extracellular ion flow	
**Neural organisms**	Exhibit a highly conserved oscillation frequency structure	Oscillations arise from intrinsic pacemaker activity of neurons
	SELFOs may emerge from a “hierarchy of pacemakers”	Specific oscillation patterns depend on neural circuit architecture
	SELFOs may coordinate and constrain lower-level components	

Although these systems share certain similarities, it is important to note that the firing oscillations they display reflect significant differences (Table 1). Variable mechanisms produce the oscillations, including ion flux through membrane channels in nonneural cells, which influences protein conductivity, intrinsic pacemaker activity in neurons, which induces oscillations in neural cells. Additionally, the functions and oscillation patterns of SELFOs may vary between neural circuit architectures and organisms.

## From Chaos to Self-Organisation: The Principles Governing Spontaneous Oscillations

Spontaneous oscillations are a characteristic feature of self-organisation in complex systems. They arise from the complicated interaction of multiple components without any external force pushing them. These oscillations occur in nonlinear, open systems that are far distant from thermodynamic equilibrium. In these systems, a continuous flow of energy and matter allows the system to sustain a dynamic steady state [45, 46]. Spontaneous oscillations can be detected in various biological and chemical systems, such as the rhythmic contractions of the heart [47] and the periodic chemical reactions in the Belousov–Zhabotinsky system [48, 49].

Complex interactions between the components of kombucha and proteinoids can give rise to spontaneous oscillations in these systems. Kombucha, a beverage made from fermented tea, contains a symbiotic culture of bacteria and yeast (SCOBY) that undergoes a series of biochemical processes during the fermentation process. The SCOBY’s metabolic processes, together with the chemical changes of the tea components, have the ability to cause oscillations in certain factors, such as pH, redox potential, or concentrations of particular metabolites [50–54].

Proteinoids, which are amphiphilic polypeptides generated under prebiotic conditions, have been demonstrated to display complex behaviours, such as self-assembly and catalytic activity [55–58]. The assembly and interactions of these polypeptides can lead to oscillatory dynamics in the concentrations of different molecular species or in the physical characteristics of the system, such as surface tension or viscosity [59–61].

The occurrence of self-generated oscillations in these systems can be comprehended by using the principles of nonlinear dynamics and the theory of dissipative structures [62]. Small disturbances in nonlinear systems have the potential to be magnified, resulting in the destabilisation of the equilibrium state and the formation of new, organised patterns. The maintenance of these structures is achieved through the waste of energy, which causes the system to move away from equilibrium and allows the oscillatory behaviour to continue.

Experimental and theoretical approaches can be used to analyse the principles that regulate spontaneous oscillations in kombucha and proteinoids. Through experimental means, the rhythmic dynamics of these systems can be better understood by doing time-resolved measurements of important variables, such as pH, redox potential, or concentrations of essential metabolites. Microscopic observations can be used to enhance these data and study the spatial arrangement and temporal changes of the system’s components.

Mathematical models can be created using ordinary or partial differential equations to describe the fundamental processes and interactions in kombucha and proteinoids. These models have the ability to include the pertinent physical, chemical, and biological mechanisms, such as reaction kinetics, diffusion, and self-assembly. An examination of bifurcation analysis and numerical simulations of these models can uncover the circumstances under which spontaneous oscillations arise and the elements that impact their properties, including amplitude, frequency, and stability.

Comprehending the underlying principles that control spontaneous oscillations in kombucha and proteinoids not only provides insight into the fundamental mechanisms of self-organisation in complex systems but also has practical implications for the advancement of innovative biomaterials and the creation of synthetic biological systems. By utilising the principles of spontaneous oscillations, scientists have the potential to fabricate materials with distinct characteristics or manipulate living systems to achieve specific functions, such as precise drug administration or biological sensing.

### Electrostatic Interactions and Material Flow Equilibration: The Emergence of Electrical Excitability in Proteinoid Microspheres

Matsuno et al. [63] proposed that the electrostatic interactions between basic and acidic proteinoids are the origin of spontaneous oscillations in proteinoid microspheres. Electricity can be excited in these microspheres because of a process called material flow equilibration. This process keeps the flow of material steady while the microspheres are self-assembling. Matsuno et al.’s theoretical study shows that the interaction rate coefficients that measure how strongly basic and acidic proteinoids are coupled have a unique property that causes the membrane potential to spike in a saw-tooth pattern. The intake flow of basic proteinoid B^+^ from the outside of the microsphere per unit time is given by

1 $${h}_{B}^{\left(in\right)}={k}_{1}\left({n}_{A}-{n}_{B}\right)$$

where *n*_*A*_ and *n*_*B*_ are the numbers of acidic and basic proteinoid molecules inside the microsphere, respectively, and *k*_1_ is the interaction rate coefficient per molecule of acidic proteinoid.

The outflow of basic proteinoid B^+^ from the microsphere S per unit time is:

2 $${h}_{B}^{\left(out\right)}={k}_{2}{n}_{B}$$

where *k*_2_ is the corresponding rate coefficient per molecule of basic proteinoid.

The dynamics of the proteinoid microspheres can be described by the following equations:

3 $$\frac{d}{dt}{k}_{1}\left({n}_{A}-{n}_{B}\right)-\frac{d}{dt}{k}_{2}{n}_{B}-2\left({k}_{1}+{k}_{2}\right)\frac{{dn}_{B}}{dt}=0$$

4 $$\frac{{dn}_{B}}{dt}=\frac{1}{2}\frac{{dk}_{1}}{dt}\left({n}_{A}-{n}_{B}\right)-\frac{1}{2}\frac{{dk}_{2}}{dt}{n}_{B}$$

where *k*_1_ and *k*_2_ are the interaction rate coefficients, *n*_*A*_ and *n*_*B*_ are the numbers of acidic and basic proteinoid molecules inside the microsphere, respectively. Equations (3) and (4) characterise the process of material flow equilibration, so providing the realisation of material flow continuity. The instability of the stationary state *h*_*B*_ = 0 leads to the emergence of spontaneous oscillations in the proteinoid microspheres, characterised by a set of inequalities:

5 $${h}_{B}>0 \text{and }\frac{{dn}_{B}}{dt}>0$$

or

6 $${h}_{B}<0 \text{and }\frac{{dn}_{B}}{dt}<0$$

The instability of the stationary state *dn*_*B*_*/dt* = 0 leads to the emergence of spontaneous oscillations in the proteinoid microspheres.

**Fig. 1:**
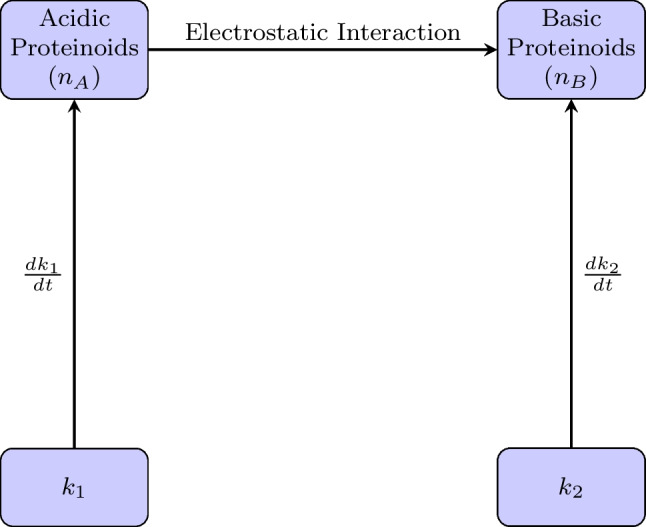
Schematic representation of the dynamics of proteinoid microspheres, as described by Matsuno’s equations. The electrostatic interaction between acidic (*n*_*A*_) and basic (*n*_*B*_) proteinoids is governed by the interaction rate coefficients *k*_1_ and *k*_2_. The equations ensure material flow equilibrium and the emergence of spontaneous oscillations

The dynamics of proteinoid microspheres, as proposed by Matsuno in 1984 [63], can be illustrated by the schematic representation shown in Fig. 1. The figure depicts the electrostatic interaction between acidic (*n*_*A*_) and basic (*n*_*B*_) proteinoids, governed by the interaction rate coefficients *k*_1_ and *k*_2_.

**Fig. 2:**
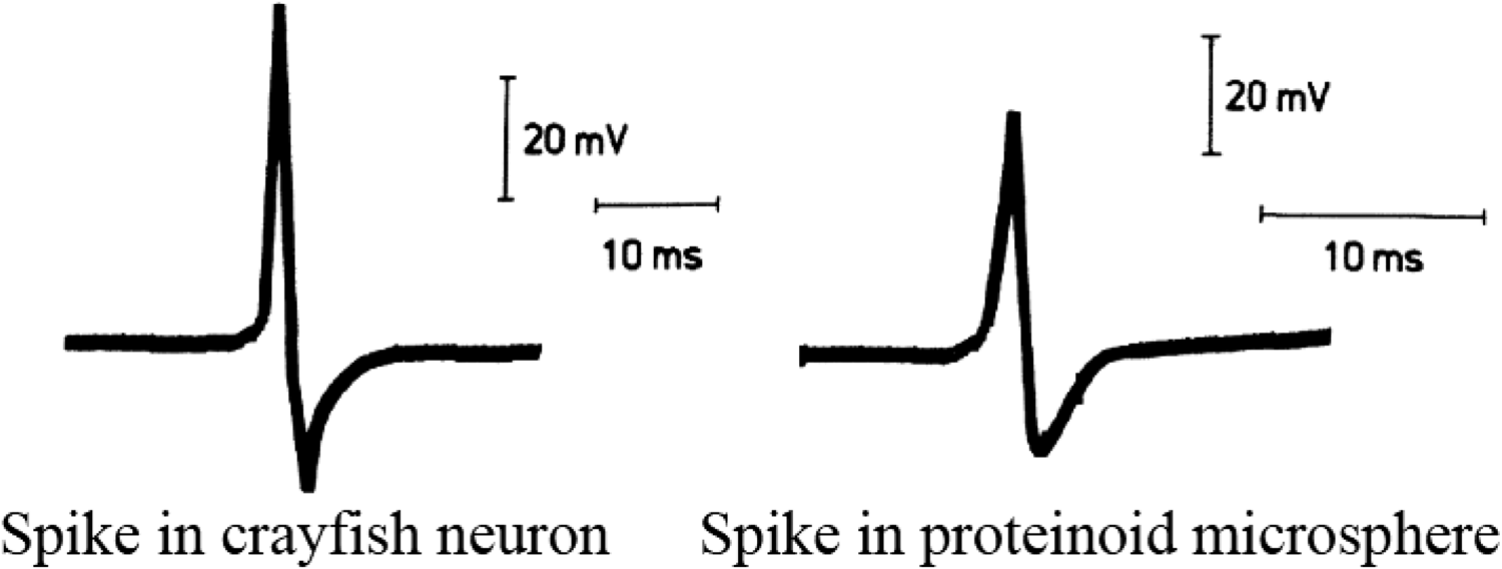
Action potential resembling that of a neuron. (Left) spiking in crayfish stretch receptor neuron. (Right) spiking in microsphere of 2:2:1-proteinoid [64]

The artificial cells, obtained through the self-organisation of thermal proteins, exhibit remarkable similarities to modern cells, including their electrical properties. Figure 2 illustrates the striking resemblance between the action potential of a crayfish stretch receptor neuron and the spiking behaviour observed in a microsphere composed of a 2:2:1 proteinoid. This similarity suggests that artificial cells, produced by retracing the evolutionary pathway, can serve as models for understanding the emergence and properties of neurons [64]. The extensive research conducted over the past three decades has not only catalogued the properties of these artificial cells but also provided insights into their potential role as artificial neurons [65–68]. Researchers consider these cells as artificial neurons due to their electrical behaviour and connectional properties [68–70]. This perspective opens up the possibility of constructing or engineering an artificial brain using the self-organising properties of thermal proteins, mimicking the processes employed by evolutionary mechanisms. We can approach the resolution of the mind–body problem by recognising that thermal proteins not only give rise to cells, but also produce excitable cells. When the protein composition is appropriate, the cells exhibit the characteristics of neurons. The appearance of thermal proteins on Earth, starting with informed proteins, led directly to the emergence of neurons, which then evolved through self-association into brains. This simplified and unifying picture serves as a foundation for comprehending the origins of neural systems.

The spontaneous formation of amino acid sequences, starting from the amino acids themselves, is a form of self-organisation coupled with bond formation. This unique process is made possible by the distinctive properties and reactions of amino acids, which belong to a family of molecules unparalleled in nature. The twenty common amino acid types are sibling molecules, sharing the family imprint of amino and acid groups while being differentiated by their characteristic side chains. The amino and acid groups participate in peptide bond formation, while the side chains contribute to the coupling rates between individual amino acids and the growing peptide chain.

**Fig. 3:**
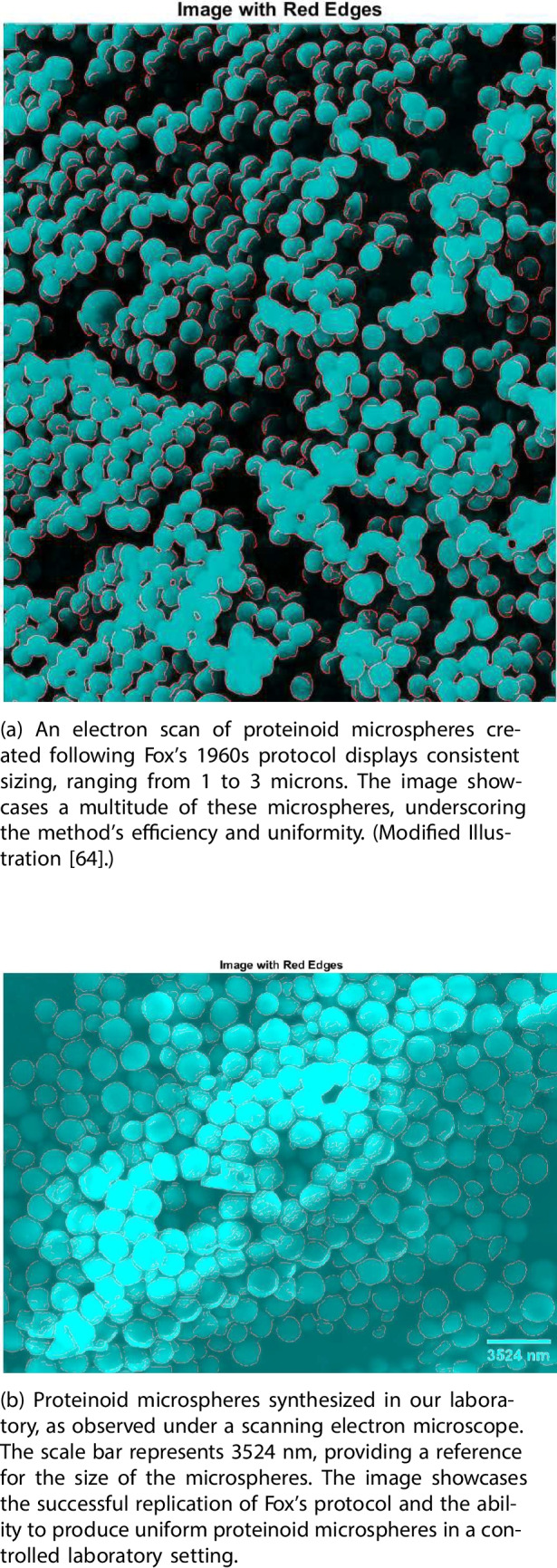
Scanning electron micrographs of proteinoid microspheres synthesised using Fox’s protocol from the 1960s (above) and in our laboratory (below), illustrating the uniformity and abundance of these structures, with diameters ranging from 1 to 3 microns

Figure 3a (above) and Fig. 3b (below) showcase the remarkable uniformity and abundance of proteinoid microspheres synthesised using Fox’s protocol from the 1960s and in our laboratory, respectively. These scanning electronmicrographs reveal the successful formation of microspheres with diameters ranging from 1 to 3 microns, demonstrating the efficiency and consistency of the production method.

The peptides formed through amino acid sequencing manifest their attractions by organising themselves into cells through intramacromolecular binding, as discovered by Fox and Harada [65]. This crucial step involves the transmission of information through the pulling inward of parts of the peptide molecule, driven by the attractions between amino acids largely fixed in larger molecules. The tendency of polyamino acid molecules to self-organise is believed to be at the core of the original cell formation [71]. This expression of information, illustrated in Fig. 3, has not been extensively studied and offers significant promise for further investigation.

**Fig. 4:**
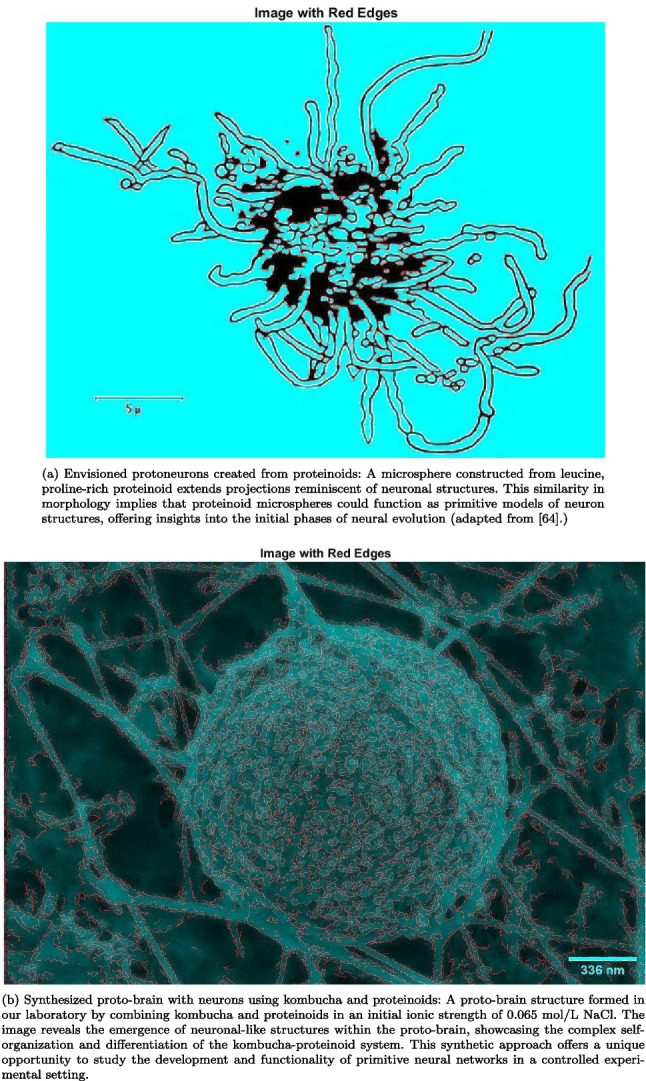
This figure highlights the self-organising properties of proteinoid-based systems and their relevance in understanding the early stages of neural evolution and the development of primitive neural networks

The emergence of complex structures and behaviours from simple building blocks is a hallmark of self-organising systems. In order to figure out where neural networks come from and how they have changed over time, proteinoid microspheres and kombucha-proteinoid systems are great ways to study how neuronal-like structures form on their own. Figure 4a depicts a microsphere made from leucine, a proline-rich proteinoid, which projects outgrowths resembling those from neurons. Because of this morphological similarity, proteinoid microspheres might be able to be used as basic models of neuronal structures, helping us understand how neurons evolved in the beginning.

To learn more about how proteinoid-based systems can self-organise, we made a proto-brain structure with kombucha and proteinoids in a solution with 0.065 mol/L NaCl. This is shown in Fig. 4b. The image reveals the emergence of neuronal-like structures within the proto-brain, showcasing the complex self-organisation and differentiation of the kombucha-proteinoid system. This synthetic approach allows us to study the development and functionality of primitive neural networks in a controlled experimental setting.

The interplay between the self-assembly of proteinoids and the metabolic processes within the kombucha leads to the spontaneous formation of these neuronal-like structures. The medium’s initial ionic strength is critical in facilitating the system’s self-organisation because it influences electrostatic interactions and protein folding. The resulting structures exhibit a remarkable degree of complexity and organisation, reminiscent of the early stages of neural development.

By combining the self-organising properties of proteinoids with the metabolic richness of kombucha, we have created a unique platform for investigating the emergence of order from chaos in the context of neural evolution. The neuronal-like structures seen in Fig. 4b show that this method can be used to make basic neural networks that can help us figure out how more complex neural systems started and how they evolved.

## Exploring the Inherent Patterns of Kombucha: Identifying Oscillations

The electrical resistance dynamics of kombucha zoogleal mats exhibit fascinating rhythmic patterns that provide insights into the complex interconnections within this symbiotic community. Adamatzky et al. noted resistance spikes resembling action potentials, characterised by an average duration of 1.8 min and an amplitude of 2.2 kOhm [72]. These oscillations emerge at a significantly greater frequency than the previously documented glycolytic oscillations in yeast, which generally exhibit periods spanning hours [73], indicating a unique underlying mechanism particular to the kombucha environment.

The fluctuations in electrical resistance, between 0.13 and 0.19 MOhm, suggest a dynamic equilibrium inside the kombucha mat [72]. This spectrum of resistance levels probably indicates the dynamic metabolic condition of the microbial community, shaped by variables such as nutrition accessibility, pH variations, and interspecies interactions. The identified patterns may signify a mode of electrical communication or coordination among the various microbial populations inside the mat, similar to the electrical signalling noted in bacterial biofilms [12].

The identification of resistance spike trains in kombucha mats reveals an interesting similarity to neural firing patterns. The average of 2.4 spikes per train and a median of 2 indicate a possible capacity for information encoding within the microbial population [72]. This discovery corresponds with recent studies on microbial intelligence and collective behaviour, wherein precise signalling pathways facilitate coordinated reactions to external stimuli [74].

The spatial arrangement of the kombucha mat may significantly influence the reported resistance oscillations. The cellulose matrix produced by the symbiotic community establishes a structured environment that may promote the transmission of electrical signals. This three-dimensional architecture may function as a biological circuit board, facilitating the creation of complex electrical behaviours comparable to those observed in neuromorphic computing systems [75].

The possible correlation between electrical resistance spikes and propagating waves of depolarisation in the kombucha mat presents new opportunities for understanding microbial community dynamics. These waves, maybe initiated by metabolically driven potassium release, may function as a mechanism for long-distance communication inside the mat [72]. This effect resembles the electrical signalling found in bacterial biofilms, where ion channel-mediated communication facilitates collective behaviour [76].

The detected resistance oscillations in kombucha mats may indicate the system’s capacity to sustain homeostasis despite environmental variations. The consistent spiking pattern, averaging 20-min intervals between spikes, may signify a feedback system that regulates metabolic activities or sustains perfect conditions for the varied microbial community [72]. This self-regulatory behaviour demonstrates the exceptional plasticity of the kombucha ecosystem and its promise as a model for examining complex biological systems.

The memristive or memfractive characteristics indicated by the complex fluctuations of electrical resistance in kombucha mats present exciting opportunities for bio-inspired computing [72]. These features, defined by history-dependent resistance alterations, may serve as the foundation for innovative neuromorphic designs using living biological materials. Biohybrid systems may provide benefits regarding adaptability, self-repair, and reduced power consumption in comparison to conventional electronic systems [77].

The analysis of electrical resistance patterns in kombucha mats advances the field of bioelectricity within microbial populations. By comparing different electrically active biological systems, such as slime moulds or mycelial networks, we can elucidate the underlying principles that regulate electrical communication in simple organisms [4]. This discovery advances our knowledge of microbial ecology and facilitates novel applications in biocomputing, biosensing, and the creation of living materials with programmable electrical characteristics. Table 2 summarises the different mechanisms of spontaneous oscillations that may be involved in the complex behaviour of kombucha zoogleal mats. These mechanisms cover a wide variety of processes, including metabolic triggering, active signal propagation, and quorum sensing. The potential role of potassium channels in coordinating metabolic activity across the mat, analogous to the YugO channel observed in *B. subtilis* biofilms, is of particular interest [12]. The fermentation processes that are distinctive to kombucha may be significantly influenced by the interplay between extracellular ion fluctuations, particularly potassium, and membrane potential variations [76]. Although these mechanisms are hypothetical in the context of kombucha systems, they offer a framework for understanding the potential self-organising principles that may exist within these complex microbial communities.

**Table 2:** Potential mechanisms of spontaneous oscillations in kombucha zoogleal mats. This table defines potential pathways for spontaneous oscillations in kombucha zoogleal mats, derived from research on bacterial biofilms [12]. The activation of ion channels, especially potassium channels, is likely essential for electrical signalling inside the mat. Metabolic signals, such nutritional availability, could cause oscillations, whereas alterations in membrane potential probably mirror and affect the metabolic condition of the mat. Fluctuations in extracellular ions, particularly potassium, may influence fermentation processes. The capacity to actively transmit signals (without attenuation) throughout the population may facilitate synchronisation in extensive mats. Metabolic interaction across diverse regions or species within the mat may equilibrate numerous functions. pH-driven oscillations may be particularly significant in modulating acidity during kombucha fermentation. Quorum sensing methods may regulate biofilm production and maintenance

Mechanism	Description	Potential role in kombucha
Ion channel activity	Potassium channels (e.g. YugO in *B. subtilis*) mediate electrical signalling	Coordinating metabolic activity across the mat
Metabolic triggering	Nutrient limitation (e.g. glutamate) initiates oscillations	Responding to changes in tea composition
Membrane potential changes	Oscillations in membrane potential linked to metabolic state	Signalling between yeast and bacteria
Extracellular ion fluctuations	Periodic release and uptake of ions (especially K^+^)	Modulating fermentation processes
Active signal propagation	Nondecaying signal transmission across the community	Synchronising activity in large mats
Metabolic co-dependence	Long-range interactions between different regions of the community	Balancing activities of different microbial species
pH-driven oscillations	Changes in proton concentration affecting cellular processes	Regulating acidity during fermentation
Quorum sensing	Cell-density dependent signalling	Coordinating biofilm formation and maintenance

## Proteinoids: A Primordial Soup of Spontaneous Oscillations

The concept of spontaneous generation, which suggests that life could emerge from nonliving matter, has a rich and glorious past in the realm of scientific inquiry. The concept that living organisms could emerge from nonliving materials was first proposed by Aristotle in the fourth century BCE. This idea continued to exist for centuries [78]. Although deeply flawed in its original form, this concept established the foundation for subsequent study of the origins of life.

During the 1920s, the independent research of Oparin and Haldane initiated the development of the current scientific method for studying abiogenesis [79]. The coacervate hypothesis, initially postulated by Oparin in 1938, posited that organic compounds have the ability to form precipitate droplets in aqueous solutions, therefore potentially acting as predecessors to the development of cellular life. Haldane proposed that the first ocean, exposed to ultraviolet radiation, might have had appeared as a “hot dilute soup” where organic molecules may have arranged into increasingly complex structures [80].

The Oparin-Haldane hypothesis received significant empirical validation with the pioneering Miller-Urey experiment conducted in 1953. The synthesis of basic organic compounds, such as amino acids, from inorganic precursors was shown by Miller and Urey under conditions believed to replicate the atmospheric conditions of the early Earth [81]. The completed experiment not only presented empirical support for the feasibility of abiogenesis but also initiated an entirely new phase of investigation into prebiotic chemistry.

Expanding upon these fundamental principles, Sidney Fox and his colleagues made significant advances in the 1960s through their research on proteinoids. Fox [82] showed that amino acids, when subjected to dry heat, had the ability to spontaneously form molecules resembling proteins. The proteinoids had various interesting characteristics, such as the capacity to generate microspheres that resembled early cellular architectural forms. Fox’s research proposed a possible route from basic organic compounds to much more complex, lifelike systems.

Furthermore, Fox and Yuyama noted that proteinoid microspheres displayed specific characteristics similar to those of living cells, such as expansion, division, and even basic metabolic processes [83]. These findings prompted discussion on the possible function of proteinoids in the genesis of life, with a few researchers suggesting that they could serve as a pivotal intermediary stage between abiotic chemistry and the initial truly living cells.

A particularly fascinating feature of this research was the emergence of the concept of spontaneous oscillations in proteinoid systems. Under certain conditions, scientists noted that proteinoid microspheres might display periodic variations in size or composition, similar to the oscillatory patterns observed in certain biological systems [64]. The presence of these spontaneous oscillations indicated the potential for self-organising behaviour in prebiotic systems, which is a crucial characteristic for the initial development of life.

There has been considerable debate around the investigation of proteinoids and their possible role in abiogenesis. Critics contend that the laboratory conditions employed to produce proteinoids may not precisely resemble those of the early Earth and that the significance of these structures in relation to the true genesis of life remains unclear [84]. However, research on proteinoids has made a substantial contribution to our knowledge of the possible routes from basic chemical compounds to more complex, functional systems.

Advancements in analytical techniques and a rising interest in a discussion of artificial life have significantly revived research on proteinoids and other prebiotic systems in recent years. Recent studies systematically investigate the inherent ability of proteinoid systems to organise themselves, their capacity for storing and transmitting information, and their potential contribution to the development of early metabolic networks [85]. Although there are still unresolved problems, the investigation of proteinoids maintains its significance in elucidating the chemical and physical mechanisms that perhaps contributed to the emergence of life on Earth.

The various mechanisms responsible for spontaneous oscillations in proteinoid systems are summarised in Table 3. This detailed table demonstrates the complexity and diversity of processes that can result in oscillatory behaviour in these primordial structures. The table illustrates that these mechanisms encompass basic physicochemical processes, including pH-driven oscillations and thermal cycling, as well as more complex phenomena such as catalytic feedback loops and reaction–diffusion mechanisms. The pH-induced oscillations, initially identified by Ishima et al. [86], illustrate how minor alterations in the proteinoid’s chemical environment can result in periodic structural transformations. Conversely, reaction–diffusion mechanisms, based on Turing’s foundational research [87], propose that spatial patterns might spontaneously arise in proteinoid systems, potentially reproducing primordial morphogenetic processes. The method of autocatalytic growth and division [88, 89] is particularly noteworthy, suggesting how these embryonic proteinoid structures may have exhibited basic life-like behaviours. The association with external rhythms [90, 91] exemplifies how proteinoid systems may synchronise with environmental cycles, an essential capability for primitive life forms. These mechanisms essentially highlight the complex dynamical behaviour of proteinoid systems and their potential significance in understanding the origins of life from nonliving materials. The variety of these mechanisms, as illustrated in Table 3, indicates that spontaneous oscillations in proteinoids may have contributed to various aspects of prebiotic evolution, ranging from the initiation of basic metabolic cycles to the promotion of complex mechanisms of self-organisation and replication.

**Table 3:** Main mechanisms of spontaneous oscillations in proteinoids

Mechanism	Description	References
pH-driven oscillations	Cyclic changes in protonation state of amino acid residues lead to conformational changes and swelling/deswelling of proteinoid microspheres	[86, 92]
Thermal cycling	Temperature fluctuations cause periodic assembly and disassembly of proteinoid structures	[88]
Catalytic feedback loops	Proteinoid structures catalyse reactions that produce or consume their own components, leading to oscillatory behaviour	[91, 93]
Ion exchange	Periodic uptake and release of ions by proteinoid microspheres result in oscillatory volume changes	[57, 63]
Phase transitions	Cyclic transitions between different structural phases of proteinoids cause oscillatory behaviour	[63, 94]
Reaction–diffusion mechanisms	Spatial and temporal patterns emerge from the interplay of reaction kinetics and diffusion processes within proteinoid systems	[87, 95]
Autocatalytic growth and division	Proteinoid microspheres undergo cycles of growth and division, leading to population-level oscillations	[89, 96]
Coupling with external rhythms	Proteinoid systems synchronise with external periodic stimuli, such as light–dark cycles or tidal patterns	[90, 97]

The pH-driven oscillations in proteinoid microspheres, as described by Ishima et al. [86], can be modelled using a simple differential equation:

7 $$\frac{dV}{dt}={k}_{1}\left(\left[{H}^{+}\right]-{\left[{H}^{+}\right]}_{\text{eq}}\right)-{k}_{2}\left(V-{V}_{eq}\right)$$

where *V* is the volume of the microsphere, [*H*^+^] is the proton concentration, [*H*^+^]_*eq*_ is the equilibrium proton concentration, *V*_*eq*_ is the equilibrium volume, and *k*_1_ and *k*_2_ are rate constants. This equation summarises the fundamental relationship between fluctuations in protonation state and volume oscillations.

Thermal cycling, another fundamental mechanism of proteinoid oscillations [88], can be described using an Arrhenius-type equation for the rate of proteinoid assembly:

8 $$k={Ae}^{-E}a/RT$$

where *k* is the rate constant, *A* is the pre-exponential factor, *E*_*a*_ is the activation energy, *R* is the gas constant, and *T* is the temperature. As temperature fluctuates, the assembly and disassembly rates change, leading to oscillatory behaviour.

The catalytic feedback loops observed in proteinoid systems [91] can be represented by a set of coupled differential equations:

9 $$\frac{dX}{dt}={k}_{1}A-{k}_{2}X+{k}_{3}{X}^{2}Y-{k}_{4}X$$

10 $$\frac{dY}{dt}={k}_{5}B-{k}_{6}Y-{k}_{3}{X}^{2}Y$$

where *X* and *Y* are the concentrations of two key components, *A* and *B* are their respective precursors, and *k*_1_ through *k*_6_ are rate constants. The term *k*_3_*X*^2^*Y* represents the autocatalytic production of *X*.

For ion exchange mechanisms [57], a simple model can be constructed using the Nernst-Planck equation:

11 $${J}_{i}=-{D}_{i}\left(\frac{{\partial c}_{i}}{\partial x}+\frac{{Z}_{i}F}{RT}{c}_{i}\frac{\partial \phi }{\partial x}\right)$$

where *J*_*i*_ is the flux of ion species *i*, *D*_*i*_ is the diffusion coefficient, *c*_*i*_ is the concentration, *z*_*i*_ is the charge number, *F* is the Faraday constant, *R* is the gas constant, *T* is temperature, and *ϕ* is the electric potential. Oscillations arise from the periodic influx and efflux of ions.

The phase transitions in proteinoid systems [63] can be described using a Landau-type free energy equation:

12 $$F={F}_{0}+a\left(T-{T}_{C}\right){\psi }^{2}+{b\psi }^{4}$$

where *F* is the free energy, *F*_0_ is the base free energy, *T* is temperature, *T*_*c*_ is the critical temperature, *ψ* is the order parameter, and *a* and *b* are constants. The system oscillates between different phases as conditions change.

Reaction–diffusion mechanisms, inspired by Turing’s work [87], can be modelled using partial differential equations:

13 $$\frac{\partial u}{\partial t}={D}_{u}{\nabla }^{2}u+f\left(u, v\right)$$

14 $$\frac{\partial u}{\partial t}={D}_{u}{\nabla }^{2}u+g\left(u, v\right)$$

where *u* and *v* are the concentrations of two species, *D*_*u*_ and *D*_*v*_ are their diffusion coefficients, and *f* and *g* are reaction terms. These equations can produce spatiotemporal oscillations and patterns.

The autocatalytic growth and division of proteinoid microspheres [88] can be described by a logistic growth equation with a division term:

15 $$\frac{dN}{dt}=rN\left(1-\frac{N}{K}\right)-\alpha N$$

where *N* is the number of microspheres, *r* is the growth rate, *K* is the carrying capacity, and *α* is the division rate.

This equation can produce oscillations in the population size.

Finally, the coupling of proteinoid systems with external rhythms [90] can be modelled using a forced oscillator equation:

16 $$\frac{{d}^{2}x}{{dt}^{2}}+{\omega }_{0}^{2}x={F}_{0}\text{cos}\left(\omega t\right)$$

where *x* is the system’s state variable, *ω*_0_ is the natural frequency of the system, *F*_0_ is the amplitude of the external force, and *ω* is the frequency of the external rhythm. This equation describes how the proteinoid system can synchronise with external periodic stimuli.

**Fig. 5:**
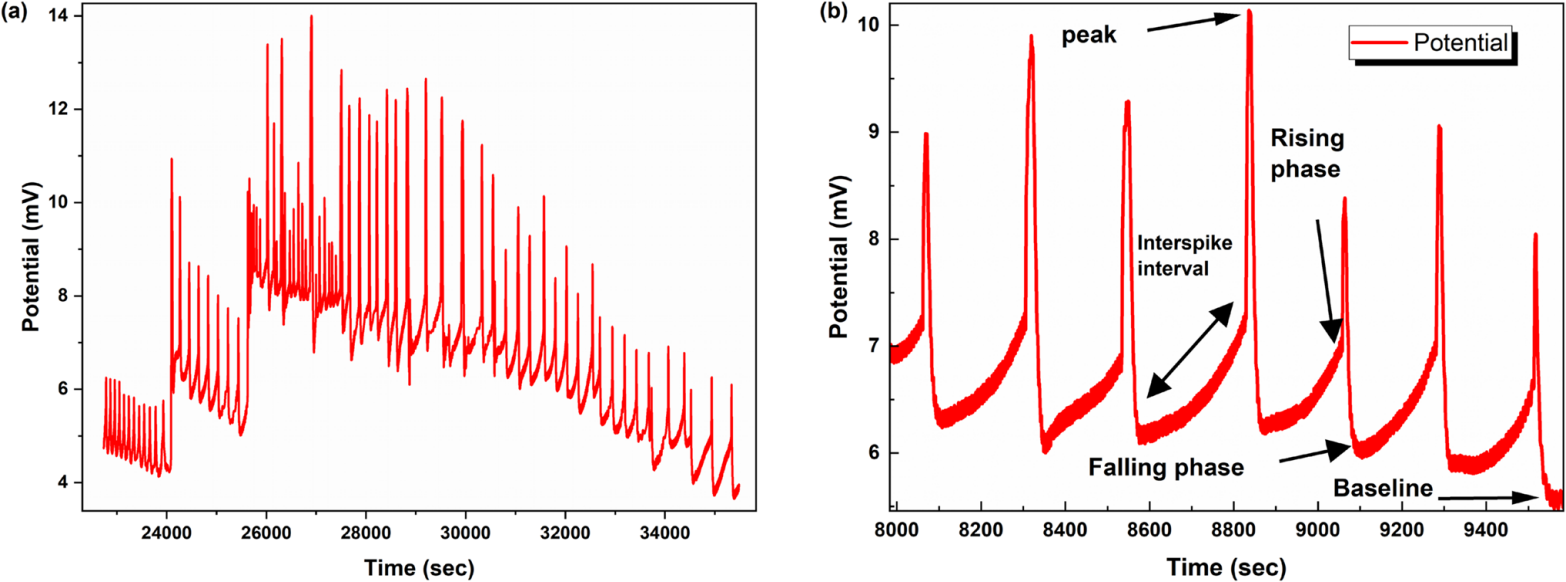
Electrical potential measurements of L-Glu:L-Phe:PLLA proteinoid. **a** The entire spectra activity over an extended time period, showing the overall pattern of electrical potential fluctuations. The graph demonstrates a complex series of spikes with varying amplitudes, suggesting a dynamic and responsive system. **b** An enlarged region of the spectra, highlighting the detailed characteristics of individual spikes. Key features are annotated, including the peak, rising phase, falling phase, interspike interval, and baseline. This detailed view reveals the consistent pattern of rapid depolarization followed by repolarization, reminiscent of action potentials in biological systems. The graph shows a series of seven distinct spikes over a time course of approximately 1400 s. Each spike is characterised by a rapid rising phase, a sharp peak (reaching potentials of 8–10 mV), followed by a steep falling phase that overshoots the baseline before gradually returning to it. The interspike interval, measuring about 200–300 s, demonstrates a regular firing pattern, suggesting a stable underlying oscillatory mechanism in proteinoid. The baseline potential, around 6 mV, shows slight variations between spikes, which could indicate subtle changes in the proteinoid’s resting state or environmental conditions. The amplitude and shape of the spikes remain relatively consistent throughout the recording, implying a robust and repeatable process within these proteinoid systems The regular spiking pattern suggests an intrinsic oscillatory mechanism within the proteinoid structure, potentially mimicking primitive cellular signalling processes. These electrical behaviours in proteinoid systems provide insights into possible prebiotic mechanisms that could have led to the development of more complex biological signalling pathways. Adapted from [94]

Figure 5 illustrates the unique similarities between the electrical behaviour of L-Glu:L-Phe:PLLA proteinoids and the action potentials observed in biological systems. This figure provides an in-depth review of the electrical potential measurements. Figure 5a displays the entire spectra activity, while Fig. 5b concentrates on an enlarged region to emphasise specific spike characteristics. Figure 5a illustrates the proteinoid system’s complex series of potential fluctuations, which are distinguished by distinct spikes that occur at regular intervals. Upon closer examination (Fig. 5b), each spike exhibits a consistent pattern of rapid depolarisation followed by repolarisation, which is consistent with the primary characteristics of neuronal action potentials. The pulses, which have amplitudes of 8–10 mV, are remarkably regular, occurring every 200–300 s. This suggests that the proteinoid structure contains an intrinsic oscillatory mechanism. The robust and repeatable process that could be analogous to primitive cellular signalling is suggested by the consistent spike morphology, which features rapid rising phases, sharp peaks, and steep falling phases, in conjunction with the regular firing pattern. The dynamic nature of the proteinoid’s resting state is further suggested by the baseline potential of approximately 6 mV, which exhibits minor variations between spikes. Mougkogiannis et al. [94] have reported that these observations offer compelling evidence for the potential role of proteinoids in the development of prebiotic signalling mechanisms. This information provides valuable insights into the potential origins of more complex biological communication systems.

## Spontaneous Oscillations: A Key to Unlocking Life’s Origins?

The realisation of self-sustaining chemical oscillations presents a fascinating connection between nonliving and living systems, with significant implications for theories on the origin of life. The proteinoid microsphere model proposed by Fox offers a strong foundation for investigating the origins of oscillatory behaviours in prebiotic systems [98].

Proteinoid microspheres demonstrate various characteristics that resemble those of early cells, such as selective membranes, catalytic activities, and the capacity to grow and divide [99]. One important question to consider is whether these protocellular structures could have developed inherent oscillatory dynamics that could drive early metabolic cycles. These oscillations may have served as a foundation for basic timekeeping mechanisms and coordinated chemical processes, which are characteristic features of living systems.

The observed catalytic activities in proteinoid systems, such as oxidation–reduction reactions [100], indicate the possibility of autocatalytic feedback loops. Under suitable nonequilibrium conditions, these could potentially result in chemical oscillations similar to those observed in systems such as the Belousov-Zhabotinsky reaction [101]. The properties of proteinoid microspheres may have enhanced the compartmentalisation and coupling of oscillatory reactions.

In addition, the capacity of proteinoid microspheres to create junctions and facilitate the exchange of materials between units [70] suggests the potential for coordinated oscillations among protocell populations. This collective behaviour may have facilitated the development of complex and coordinated chemical dynamics at a supracellular level. Although there is still a need for direct experimental evidence of spontaneous oscillations in proteinoid systems, the model presents a promising opportunity to explore the origins of periodic behaviours crucial to life. Further research that integrates proteinoid chemistry with nonequilibrium dynamics has the potential to provide significant insights into this crucial phase of prebiotic evolution. Research into oscillatory phenomena in protocellular models can shed light on a fundamental transition in the history of life on Earth. It bridges the gap between simple chemical systems and the complex temporal organisation of living cells.

### The Belousov-Zhabotinsky Reaction: A Paradigm for Chemical Oscillations

The Belousov-Zhabotinsky (BZ) reaction is an essential element in the field of nonlinear chemical dynamics and provides a valuable analogy for exploring potential oscillatory behaviours in prebiotic systems [102]. Discovered by Belousov in the 1950s and later extensively studied by Zhabotinsky, this reaction showcases the ability of basic chemical components to generate complex, rhythmic behaviours that bear resemblance to biological processes [103, 104]. The BZ reaction is a well-known chemical process that involves the oxidation of an organic substrate, usually malonic acid, by bromate ions. This reaction takes place in the presence of a metal catalyst, commonly cerium or ferroin, under acidic conditions. The system displays sustained oscillations in chemical concentrations, which can be observed as periodic colour changes or, in unstirred conditions, as propagating waves and spiral patterns [105, 106].

The BZ reaction is a complex mechanism that encompasses various interconnected chemical processes, resulting in the formation of a network of feedback loops [107]. The reaction at its core alternates between two main states: one where the metal catalyst is in its reduced form, and another where it is oxidised. The transition between these states is influenced by the accumulation and reduction of crucial intermediates, specifically bromide ions and bromous acid [108]. The interaction of these processes results in a chemical “clock” that exhibits precise and consistent oscillations, sometimes lasting for extended periods of time under particular conditions [101]. Many researchers have drawn parallels between these oscillations and biological rhythms, suggesting that the periodic behaviours observed in living systems may be driven by similar mechanisms [45, 109].

The BZ reaction is known for its interesting ability to display spatiotemporal patterns when conducted in thin layers or gels [110]. The emergence of various patterns, such as target waves, spiral waves, and more complicated patterns, is a result of the interaction between oscillatory chemistry and diffusion processes [111]. These phenomena have been thoroughly investigated as models for pattern formation in biological systems, ranging from the aggregation of slime moulds to the electrical activities in cardiac tissue [87, 112]. The BZ reaction’s ability to generate and sustain complex patterns without external input has significant implications for understanding the emergence of ordered structures in prebiotic chemical systems [113].

The BZ reaction has been instrumental in advancing the field of nonlinear dynamics and chaos theory [46]. Research on this system has uncovered a wide range of dynamic phenomena, such as period-doubling bifurcations, chaos, and different types of synchronisation [48, 114]. The findings have significantly contributed to our understanding of complex chemical systems and have also shed light on the mathematical principles that govern various natural phenomena, such as weather patterns and population dynamics [115]. Within the realm of origin of life studies, the BZ reaction stands as a compelling illustration of how basic chemical principles can lead to complex, life-like phenomena, potentially closing the space between nonliving and living systems [116].

In the past few years, there have been significant advancements in employing BZ-type reactions for the development of “chemical computing” systems [117]. A study has shown that BZ reactions can be used for performing fundamental logical operations, storing information, and solving basic computational problems [118, 119]. Recent advancements suggest that oscillatory chemical systems may have contributed to the development of basic information processing capacity in early life forms [120]. In addition, the combination of BZ reactions with other chemical systems, such as protocell models like proteinoid microspheres, provides new opportunities for investigating the possible development of complex, life-like behaviours from prebiotic chemistry [121, 122]. The BZ reaction remains a crucial model system in ongoing research, stimulating the development of fresh hypotheses and experimental methods in the pursuit of understanding the chemical beginnings of life.

### Oscillatory Dynamics in Autocatalytic Networks: Precursors to Metabolism

The study of autocatalytic networks is essential for understanding the connection between basic chemical oscillations and the complex metabolic cycles found in living systems. The self-sustaining reaction networks described in this study provide a compelling model for the emergence of primitive metabolic pathways [97]. Exploring the emergence and endurance of oscillatory dynamics in autocatalytic networks can provide insights into the transition from abiotic chemical cycles to the essential rhythmic processes in biology [123].

Autocatalytic sets, initially proposed by Stuart Kauffman, consist of molecules that catalyse each other’s production from a basic food set [124]. The current study has shown that networks can display complex dynamics, such as oscillations, as both theory and experiments have revealed. As an example, Semenov et al. demonstrated that autocatalytic networks using template-directed ligation reactions can generate sustained oscillations in molecular concentrations [125]. The findings indicate a possible mechanism for the development of periodic behaviours in prebiotic chemical systems, without requiring complicated cellular machinery.

### Quantum Effects in Biological Oscillations

Recent advancements in quantum biology have provided new insights into the influence of quantum effects on biological processes, particularly oscillatory phenomena. Classical models have traditionally shaped our understanding of biological rhythms; however, recent evidence indicates that quantum coherence and entanglement could be significant factors in specific oscillatory systems at the molecular level [126]. The potential involvement of quantum effects in the avian magnetic compass represents a significant discovery in this field. Theoretical and experimental investigations indicate that light-induced radical pair reactions in cryptochrome proteins can produce quantum-coherent oscillations that respond to weak magnetic fields [127]. These oscillations may serve as a mechanism for avian navigation by enabling the detection of the Earth’s magnetic field. This finding explains an ever-present biological enigma and indicates that quantum effects may have influenced the evolution of sensory systems. Evidence has emerged for quantum coherence in energy transfer within light-harvesting complexes in the context of photosynthesis. Engel et al. employed two-dimensional electronic spectroscopy to detect long-lived quantum coherence in photosynthetic complexes at room temperature [128]. Quantum beats occurring on the femtosecond timescale may enhance the efficiency of photosynthetic energy transfer. This discovery has significant implications for our understanding of the mechanisms by which life employs light energy and indicates that quantum effects may have been used by evolution from early stages.

The potential role of quantum tunnelling in enzyme catalysis signifies a new area of exploration within quantum biology. Klinman and Kohen have provided evidence suggesting that hydrogen tunnelling may play a role in enzyme-catalysed hydrogen transfer reactions [129]. This quantum effect might improve the catalytic efficiency of specific enzymes by facilitating reactions via classically forbidden pathways. The oscillatory characteristics of quantum tunnelling events in these systems provide new insights into the origins of rhythmic behaviours in prebiotic and early biological systems. Recent theoretical investigations have examined the potential for quantum synchronisation among groups of quantum oscillators, including those that may be present in biological systems. Manzano et al. demonstrated that quantum correlations can induce synchronisation phenomena absent in classical systems [130]. Although direct experimental evidence for quantum synchronisation in biological systems is currently absent, these theoretical insights indicate interesting possibilities for the emergence of coordinated behaviour at the quantum level in early life forms.

### Stochastic Resonance in Biological Oscillators

Stochastic resonance is a phenomenon in which noise improves signal detection in nonlinear systems, and it has become an important focus of research in biological oscillations. This concept, initially introduced in climate dynamics, has been extensively applied to the study of sensory processing and cellular signalling mechanisms [131]. Stochastic resonance in biological systems may significantly amplify weak periodic signals, providing insights into the mechanisms by which early life forms detected and responded to environmental signals. Recent studies demonstrate stochastic resonance across various biological contexts, including sensory neurons and genetic networks. Douglass et al. demonstrated that mechano-receptor cells in crayfish display increased sensitivity to weak periodic signals when optimal noise levels are present [132]. This finding indicates that noise, typically viewed as harmful to biological processes, may be used by living systems to improve signal detection and information processing. Stochastic resonance has been observed in the dynamics of ion channels at the molecular level. Bezrukov and Vodyanoy showed that introducing noise can improve the detection of weak signals by voltage-dependent ion channels [133]. This mechanism may elucidate the development of early cellular systems’ sensitivity to environmental fluctuations, which could influence the evolution of more complex signalling pathways. Genetic oscillators, essential for circadian rhythms and various biological cycles, might use stochastic resonance to sustain robust oscillations in the presence of molecular noise. Vilar et al. established a theoretical framework demonstrating that noise can stabilise genetic oscillators, which may explain the resilience of circadian rhythms to variations in molecular concentrations [134]. This insight offers a novel perspective on the evolution of reliable biological timekeeping mechanisms from inherently noisy chemical systems. The concept of stochastic resonance provides valuable insights into the emergence of collective behaviour in populations of coupled oscillators. Zhou et al. illustrated that noise can facilitate synchronisation in arrays of chaotic oscillators [135]. The phenomenon of noise-induced synchronisation may serve as a mechanism for the emergence of coordinated behaviour in early multicellular systems, potentially linking individual cellular oscillations to organism-level rhythms.

### Circadian Rhythms in Unicellular Organisms: Evolutionary Insights

The examination of circadian rhythms in unicellular organisms offers significant understanding of the evolution of biological timekeeping mechanisms. Circadian clocks are extensively studied in multicellular organisms; however, their existence and role in single-celled organisms provide valuable insights into the origins of these oscillatory systems. Recent studies indicate that even the most basic organisms exhibit advanced timekeeping mechanisms, prompting a reevaluation of the key requirements for circadian rhythmicity [136]. Cyanobacteria, especially the model organism *Synechococcus elongatus*, serve as effective systems for the study of prokaryotic circadian clocks. The identification of circadian rhythms in these ancient organisms has significantly advanced our understanding of the evolutionary development of biological timekeeping. The cyanobacterial circadian oscillator can be reconstituted in vitro using only three proteins: KaiA, KaiB, and KaiC [137]. This minimalist system exhibits sustained oscillations in the phosphorylation state of KaiC over a 24-h period, illustrating how complex temporal organisation can arise from simple molecular interactions. *Chlamydomonas reinhardtii*, a unicellular alga within the eukaryotic domain, has contributed significantly to the understanding of circadian regulation in cellular processes. Research indicates that this organism has a circadian clock that regulates multiple physiological processes, such as phototaxis and cell division [138]. The circadian clock in Chlamydomonas displays similarities to those found in higher plants, yet it also possesses distinct characteristics, indicating divergent evolutionary trajectories in the development of circadian systems among various lineages.

Recent studies have identified circadian rhythms in nonphotosynthetic unicellular eukaryotes, including the budding yeast *Saccharomyces cerevisiae*. Eelderink-Chen et al. showed that yeast cells display temperature-compensated rhythms in cellular redox state, with a period of around 12 h [139]. This finding challenges the traditional perspective that circadian clocks primarily evolved in photosynthetic organisms as an adaptation to the light–dark cycle, indicating that the capacity to anticipate and respond to periodic environmental changes may be a more essential characteristic of cellular life. The examination of circadian rhythms in unicellular organisms has yielded insights into the relationship between timekeeping mechanisms and the cell cycle. Research on the dinoflagellate *Lingulodinium polyedrum* has demonstrated complex interactions between the circadian clock and cell division cycles [140]. The findings indicate that the incorporation of temporal information with fundamental cellular processes may have been an early characteristic in the evolution of biological timekeeping, possibly affecting the formation of more complex regulatory networks in multicellular organisms.

### Oscillatory Dynamics in Synthetic Biology

Synthetic biology has advanced in engineering artificial oscillatory systems, yielding insights into the design principles of biological rhythms. Synthetic oscillators function as important instruments for elucidating natural biological clocks and present potential applications in biotechnology and medicine. Researchers have constructed and manipulated artificial genetic circuits to create various oscillatory behaviours that replicate and expand upon those observed in nature [141]. The “repressilator”, developed by Elowitz and Leibler, represents a seminal contribution in this field [142]. The synthetic genetic oscillator, composed of three transcriptional repressors organised in a negative feedback loop, exhibited sustained oscillations in gene expression within *Escherichia coli*. The repressilator demonstrated a proof-of-concept for the engineering of complex dynamic behaviours in living cells, subsequently inspiring various modifications and enhancements in synthetic oscillator design. Researchers have advanced the development of synthetic oscillators, achieving greater robustness and tunability. Stricker et al. developed a “robust oscillator” that integrates positive and negative feedback loops, yielding more stable and tunable oscillations than the original repressilator [143]. This study emphasised the significance of network topology in influencing the characteristics of biological oscillators and offered insights into the design principles that may govern natural circadian clocks. The advancement of synthetic oscillators has extended beyond transcriptional regulation to include additional cellular processes. Fung et al. developed a post-translational oscillator using the cyclic sequestration and release of a sigma factor in *E. coli* [144]. This approach showed that oscillatory dynamics can be obtained through mechanisms beyond transcriptional regulation, expanding our understanding of potential architectures for biological timekeeping systems. Recent advancements in synthetic biology have facilitated the development of multicellular oscillators, wherein populations of engineered cells demonstrate synchronised oscillations. Danino et al. created a synthetic circuit that produces synchronised oscillations in a growing population of *E. coli* by using quorum sensing mechanisms [145]. This study elucidated the emergence of collective behaviours from interactions among individual cellular oscillators, a phenomenon important to understanding complex rhythmic behaviours in multicellular organisms and microbial communities.

The various oscillatory systems discussed in this section, ranging from simple chemical oscillations to complex biological rhythms, offer diverse perspectives on the potential origins and evolution of periodic behaviours in early life forms. Table 4 presents a comparative analysis of these systems, emphasising their essential characteristics, significance to the origins of life, and illustrative examples or models. This synthesis demonstrates the multiscale characteristics of biological oscillations and the evolution from abiotic chemical cycles to the complex temporal organisation found in living cells.

**Table 4:** Comparative analysis of oscillatory systems relevant to the origins of life. This table summarises essential characteristics of different oscillatory phenomena, emphasising their possible functions in prebiotic and early biological systems. Simple chemical oscillations, such as the Belousov-Zhabotinsky reaction, and complex biological rhythms, exemplified by circadian clocks in unicellular organisms, illustrate the emergence and evolution of periodic behaviours in early life forms. The incorporation of quantum effects and stochastic resonance demonstrates the multiscale characteristics of biological oscillations, whereas synthetic biology methodologies offer a framework for testing hypotheses regarding the design principles that govern these rhythms. These oscillatory systems provide various insights into the transition from abiotic chemical cycles to the complex temporal organisation found in living cells

Oscillatory system	Key features	Relevance to origins of life	Example/model
Proteinoid microspheres	Selective membranes, catalytic activities, growth and division	Potential for primitive metabolic cycles	Fox’s thermal proteins
Belousov-Zhabotinsky reaction	Self-sustaining chemical oscillations, pattern formation	Model for prebiotic chemical dynamics	Malonic acid oxidation by bromate
Autocatalytic networks	Self-sustaining reaction cycles	Precursors to metabolic pathways	Kauffman’s autocatalytic sets
Quantum biological oscillations	Coherence in energy transfer, tunnelling in enzymes	Potential role in early sensory systems	Cryptochrome in avian magnetoreception
Stochastic resonance	Noise-enhanced signal detection	Amplification of weak environmental signals	Ion channel dynamics
Unicellular circadian rhythms	Minimal timekeeping mechanisms	Evolution of biological clocks	Cyanobacterial Kai protein system
Synthetic oscillators	Engineered genetic circuits	Insights into design principles of biological rhythms	Repressilator in *E. coli*

**Fig. 6:**
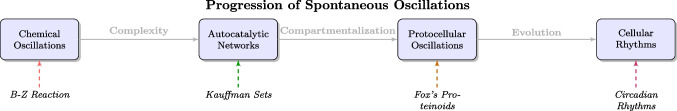
Progression of spontaneous oscillations from basic chemical systems to complex biological rhythms. This diagram depicts the escalating complexity and organisation of oscillatory phenomena that may be related to the origins of life. The exploration begins with fundamental chemical oscillations, exemplified by the Belousov-Zhabotinsky reaction, and advances through self-sustaining autocatalytic networks and protocellular systems, such as Fox’s proteinoid microspheres, resulting in the complex cellular rhythms found in living organisms, including circadian clocks. Each stage signifies an important rise in complexity and self-organisation, possibly reflecting critical transitions in the emergence of life

The transition from basic chemical oscillations to complex biological rhythms is a crucial element in understanding the origin and evolution of life. Figure 6 depicts this conceptual progression, emphasising the growing complexity and organisation of oscillatory phenomena that may be significant to the origins of life.

## Harnessing the Power of Spontaneous Oscillations: Prospects and Possibilities

Studying the causes of spontaneous oscillations in chemical and biological systems has revealed many possible uses across diverse fields. The development of novel drug delivery systems using oscillatory mechanisms is a promising area of research. Researchers have investigated oscillating chemical reactions to develop pulsatile release profiles for pharmaceuticals, which may improve therapeutic efficacy and minimise side effects [146]. These systems may offer significant advantages for medications requiring precise temporal dosing, particularly in the context of chronotherapy. Self-oscillating polymers, inspired by biological rhythms, have attracted considerable interest in materials science. Smart materials capable of autonomous mechanical oscillations without external energy input present significant potential for applications in soft robotics and adaptive structures [147]. These materials, by replicating the rhythmic contractions of biological tissues like the heartbeat, may facilitate the creation of more realistic and energy-efficient artificial muscles and actuators. Neuroscience has obtained valuable insights from the study of oscillatory phenomena. The identification of stochastic resonance in neural systems has resulted in innovative methodologies for neural prosthetics and brain-computer interfaces [148]. Researchers used the noise-enhancing characteristics of stochastic resonance to create more sensitive neural implants that can detect and transmit weak neural signals with greater accuracy. Oscillatory chemical reactions have been suggested in environmental science as a method for enhancing the efficiency of water purification systems. The Belousov-Zhabotinsky (BZ) reaction has been studied for its potential in the removal of organic pollutants via oscillatory redox processes [149]. These systems may provide benefits compared to conventional water treatment methods regarding energy efficiency and adaptability to different pollutant loads. The principles of chemical oscillations are being used in the advancement of unconventional computing paradigms. Reaction–diffusion computers employ pattern-forming chemical reactions to address specific computational problems, offering potential benefits in terms of parallelism and energy efficiency [119], although in the early stages of development these chemical computing systems may ultimately improve traditional silicon-based computers in specific applications. Engineered genetic oscillators are being investigated in synthetic biology for applications that include biosensors and therapeutic devices. Researchers have developed synthetic bacterial oscillators capable of measuring elapsed time with high precision, which may facilitate novel methods for controlled gene expression and drug release in vivo [145]. These biological clocks may serve as a foundation for the development of smart probiotics or cell-based therapies that react to specific temporal signals within the body. The investigation of oscillatory phenomena in protocells and minimal synthetic cells discusses the essential criteria for maintaining chemical oscillations in sustained environments. This research improves our understanding of life’s origins and facilitates the advancement of artificial cells exhibiting programmable dynamic behaviours [150]. Synthetic cells may function as customisable platforms for various biotechnological applications, including bioremediation and the synthesis of fine chemicals. The integration of proteinoid research and fermentation processes, as demonstrated by kombucha production, offers promising opportunities for innovative biomaterials and food technologies. Proteinoids possess the capacity to form microspheres and demonstrate catalytic activities, suggesting their potential integration into the kombucha fermentation process to improve probiotic properties or introduce novel functionalities [151]. The integration of proteinoid chemistry and the complex microbial ecology of kombucha may facilitate the creation of advanced functional foods or biomaterials exhibiting distinctive oscillatory properties, thereby connecting prebiotic chemistry with contemporary biotechnology.

**Fig. 7:**
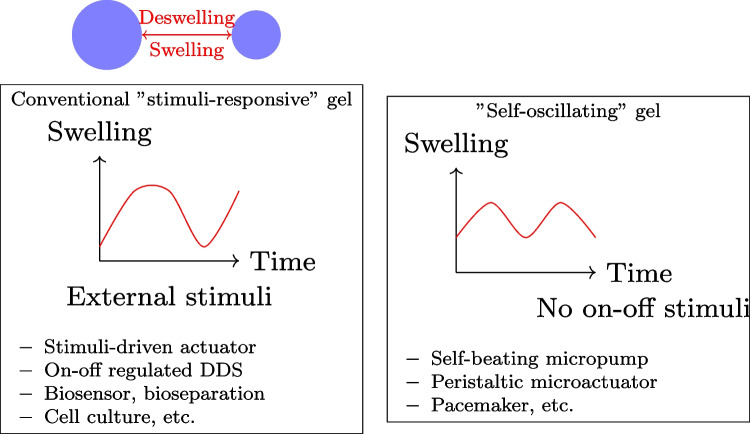
Stimulus-responsive gel and self-oscillating gel

**Fig. 8:**
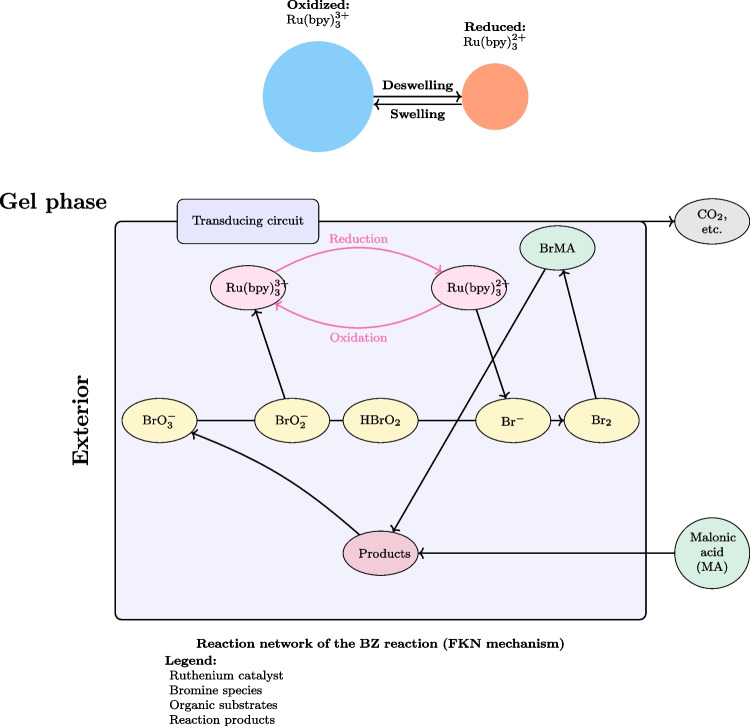
Schematic illustration of the self-oscillating polymer gel utilising the Belousov-Zhabotinsky (BZ) reaction. The upper section demonstrates the swelling-deswelling behaviour influenced by the oxidation state of the ruthenium catalyst. The lower section illustrates the complex reaction network present in the gel phase, highlighting the Field-Körös-Noyes (FKN) mechanism of the BZ reaction. Key components include the oscillating $\text{Ru}{\left(\text{bpy}\right)}_{3}^{3+}/\text{Ru}{\left(\text{bpy}\right)}_{3}^{2+}$ redox couple, various bromine species, and the organic substrate malonic acid. This intricate chemical feedback mechanism generates sustained oscillations, manifesting as periodic swelling and deswelling of the gel without external stimuli. The transducing circuit exemplifies the gel’s ability to convert chemical oscillations into mechanical motion, underscoring the remarkable self-regulating properties of this biomimetic system

Figures 7 and 8 illustrate the fundamental differences between conventional stimulus-responsive gels and self-oscillating gels, as well as the complex mechanism behind self-oscillating systems. Figure 7 highlights these two types of gels, showing that conventional gels require external stimuli to switch between swelling and deswelling states, making them suitable for applications such as stimulus-driven actuators and on–off regulated drug delivery systems [152–154]. In contrast, self-oscillating gels [155] autonomously cycle between swollen and deswollen states without external triggers, enabling their use in self-beating micropumps and peristaltic microactuators. Figure 8 examines the self-oscillating gel mechanism, highlighting its dependence on the Belousov-Zhabotinsky (BZ) reaction. This complex reaction network, following the Field-Körös-Noyes (FKN) mechanism, involves an oscillating ruthenium complex $\left(\text{Ru}{\left(\text{bpy}\right)}_{3}^{3+}/\text{Ru}{\left(\text{bpy}\right)}_{3}^{2+}\right)$ that drives the gel’s swelling-deswelling behaviour. The oxidised state $\left(\text{Ru}{\left(\text{bpy}\right)}_{3}^{3+}\right)$ corresponds to the swollen state, while the reduced state $\left(\text{Ru}{\left(\text{bpy}\right)}_{3}^{2+}\right)$ leads to deswelling. The reaction incorporates various bromine species (${\text{BrO}}_{3}^{-}$, ${\text{BrO}}_{2}^{-}$, HBrO_2_, Br^−^, Br_2_) and malonic acid as an external reactant, producing CO_2_ and other products. This self-regulating, biomimetic system effectively transduces chemical oscillations into mechanical motion, showcasing the fascinating interplay between chemistry and material science in creating autonomous, responsive materials.

## From chaos to Order: The Significance of Spontaneous Oscillations in Kombucha and Proteinoids

**Fig. 9:**
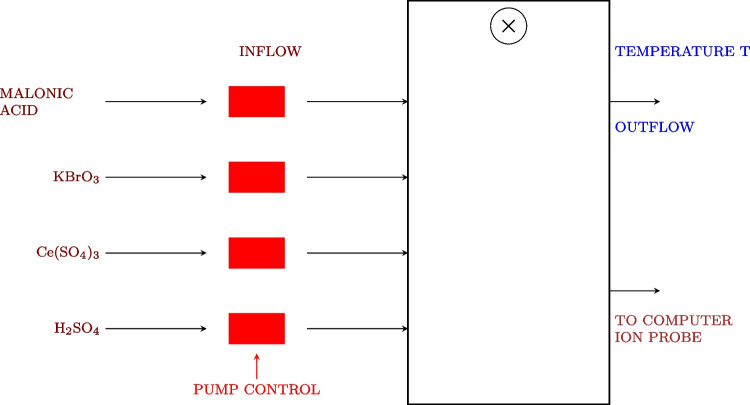
Schematic representation of a chemical reactor used to study the oscillations in the Belousov-Zhabotinsky reaction (there is a stirring device in the reactor to keep the system homogeneous). The reaction has over thirty products and intermediates. The evolution of different reaction paths depends (among others factors) on the entries controlled by the pumps

**Fig. 10:**
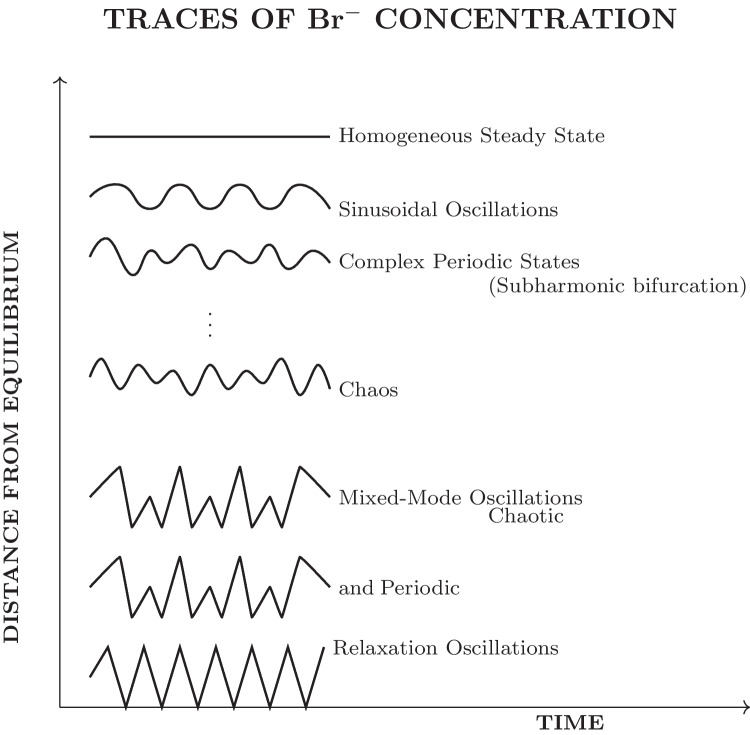
Temporal oscillations of the ion Br^*−*^ in the Belousov-Zhabotinsky reaction. The figure represents a succession of regions corresponding to qualitative differences. This is a schematic representation. The experimental data indicate the existence of much more complicated sequences

The Belousov-Zhabotinsky (BZ) reaction, depicted in Fig. 9, is fundamental to the investigation of nonequilibrium thermodynamics [156]. This chemical system illustrates the ability for self-organisation and spontaneous oscillations, thereby challenging the conventional perspective of thermodynamics that prioritised equilibrium states. In “Order Out of Chaos”, Prigogine used these systems to propose a revised perspective on the interplay between order and disorder in the natural world [13]. The complex reaction network of the BZ reaction, illustrated in Fig. 9, consists of many intermediates and feedback loops. This complex mechanism facilitates the emergence of temporal oscillations, as illustrated in Fig. 10. The oscillations exhibit a spectrum from simple periodic behaviour to complex chaotic patterns, demonstrating the diverse dynamics possible in far-from-equilibrium conditions [157]. Prigogine highlighted that systems functioning far from equilibrium have the capacity to spontaneously generate complex spatial and temporal structures [13]. Figure 10 illustrates a hierarchy of oscillatory behaviours identified in the BZ reaction, ranging from steady states to chaotic dynamics. This progression reflects Prigogine’s concept of bifurcations, wherein minor alterations in system parameters may result in qualitatively distinct behaviours [13]. The shift from sinusoidal oscillations to complex periodic states, resulting in chaos, illustrates the system’s sensitivity to initial conditions and control parameters, characteristic of nonlinear dynamics [102]. The self-oscillating polymer gel, as illustrated in Fig. 8, demonstrates the integration of chemical and mechanical processes through the BZ reaction. The coupling leads to periodic swelling and deswelling of the gel, influenced by the oxidation state variations of the ruthenium catalyst [147]. These systems connect nonliving chemical oscillators with the rhythmic behaviours seen in living organisms, a relationship that interested Prigogine and Stengers [13]. The capacity to regulate and manipulate these oscillations via external parameters, as indicated by the pump control in Fig. 9, presents opportunities for the development of responsive materials and systems. This is consistent with Prigogine’s perspective on using nonequilibrium phenomena for practical applications [158]. Precise control of reactant concentrations and temperature facilitates fine-tuning of system behaviour, potentially leading to the development of novel sensors, actuators, and adaptive materials [159]. Figure 10 illustrates the mixed-mode oscillations and chaotic behaviour, demonstrating the BZ reaction’s ability to produce complex temporal patterns. The observed patterns reflect the irregular yet structured behaviours characteristic of multiple biological systems [109]. Prigogine and Stengers posited that complexity, which emerges from straightforward foundational rules, may offer valuable insights into the origins of biological organisation and the transition from chemical systems to life [13]. One can draw parallels to other self-organising systems by examining the fermentation process in kombucha. Kombucha fermentation, although lacking the dramatic oscillations characteristic of the BZ reaction, involves complex feedback mechanisms between yeast and bacterial populations, resulting in the formation of a stable, symbiotic community [160]. This self-organisation within a biological framework reflects Prigogine’s concepts regarding dissipative structures that sustain themselves via continuous energy and matter exchange with their surroundings [13]. Proteinoids, which are thermal proteins derived from amino acid mixtures, exhibit spontaneous organisation into microspheres under specific conditions [65]. These structures, although less complex than living cells, demonstrate characteristics including growth, division, and catalytic activity. The formation of proteinoid microspheres exemplifies the emergence of order from disorder [13]. Kombucha and proteinoid systems demonstrate that complex, life-like behaviours can emerge from simple chemical or biochemical components in nonequilibrium conditions.

One remarkable illustration of spontaneous pattern generation in fluid dynamics is the Benard cell convection system. Heat applied from below can cause a thin layer of fluid to spontaneously form hexagonal convection cells, which are similar in shape to the spatial patterns seen in certain BZ reactions (Fig. 10) [161]. As the system loses energy, this self-organisation takes place, which is consistent with Prigogine’s theory of dissipative structures preserving order far from equilibrium [13]. Aggregation of slime mould provides a biological analogue to chemical oscillators such as the BZ reaction (Fig. 9). Individual *Dictyostelium discoideum* amoebae under starving conditions release periodic pulses of cyclic AMP, which cause spiral waves of chemotaxis to emerge that resemble those observed in spatially extended BZ systems [162]. A important idea in Prigogine’s work on self-organisation is shown by this process, which shows how local interactions can result in global order [158]. An interesting example of collective behaviour arising from individual oscillators is the synchronisation of firefly flashing. Fireflies have the ability to spontaneously synchronise their light outputs in large groupings, producing amazing displays [163]. Coupled oscillator systems, which are theoretically comparable to the coupling between chemical species in the BZ reaction network (Fig. 9), can be used to describe this phenomenon [164]. Another system in which order develops from seemingly random initial conditions is represented by Turing patterns, which were first proposed by Alan Turing as a mechanism for the generation of biological patterns. These patterns, which are theoretically comparable to the spatial patterns that can develop in prolonged BZ reactions, are the result of the interaction between reaction and diffusion processes [87]. Turing patterns, which represent Prigogine’s theories regarding the universal principles driving pattern formation in nature, have been seen in chemical systems and are believed to be involved in biological morphogenesis [165]. A macroscopic illustration of geological systems’ self-organisation is seen in the creation of sand dunes. Even while wind-blown sand seems random, dunes can create recurring, predictable patterns across large distances [166]. Though on a much bigger scale and slower timeline, this process of pattern generation from local interactions is similar to the emergence of spatial and temporal patterns in chemical systems such as the BZ reaction (Fig. 10). A physical illustration of self-organisation and coherent behaviour arising from noise may be seen in lasers. Similar to the BZ reaction’s transition from steady state to oscillatory behaviour (Fig. 10), a laser’s output changes from incoherent emission to coherent laser light as its power input increases [167]. This phenomenon is consistent with the theories of Prigogine regarding phase transitions and bifurcations in nonequilibrium systems [13]. One remarkable example of order emerging from complex interactions is the emergence of consciousness from brain activity. Though the precise mechanisms are still unknown, suggestions indicate that billions of neurons fire in unison to produce awareness, possibly through oscillatory behaviours similar to those in chemical systems [13]. This idea is consistent with Prigogine’s more general philosophical theories regarding the formation of complexity and novelty in nature [158]. Lastly, self-organisation at the level of collective animal behaviour is demonstrated by the establishment of social insect colonies, such as termite or ant mounds. Without centralised direction, thousands of individual insects interacting according to basic rules produce complex structures and behaviours [168, 169]. The notions of self-organisation and emergent order that are fundamental to Prigogine’s work appear in this process of collective intelligence originating from local interactions, as demonstrated by the complex behaviours that result from basic chemical interactions in systems such as the BZ reaction (Fig. 10) [13].

A well-known illustration of pattern development in nonequilibrium systems, the Rayleigh-Bénard instability reflects the concepts of self-organisation that Prigogine and Stenger [13] discusses. This phenomenon happens when heat is applied from below to a fluid layer, producing a temperature gradient that may cause convective motion.

17 $$Ra=\frac{g\beta \left({T}_{b}-{T}_{u}\right){L}^{3}}{v\alpha }$$

where *g* is gravitational acceleration, *β* is the thermal expansion coefficient, *T*_*b*_ and *T*_*u*_ are the temperatures at the bottom and top of the fluid layer respectively, *L* is the layer thickness, *ν* is the kinematic viscosity, and *α* is the thermal diffusivity [170]. When Ra exceeds a critical value, Ra_*c*_, the system transitions from a state of pure conduction to one of convection, characterised by the formation of Bénard cells. The system’s boundary conditions determine the critical Rayleigh number. ${\text{Ra}}_{c}=\frac{{27\pi }^{4}}{4}\approx 657.5$ was analytically obtained by Lord Rayleigh for the basic situation of two free boundaries [171]. Ra_*c*_ ≈ 1100*.*65 [172], however, for more realistic conditions with rigid bottom and free top bounds (such a pot of water boiled on a stove). When these boundaries are crossed, the system starts to behave in a more complicated way. Ra grows further, causing the flow to change through different regimes: time-dependent patterns, stable convection rolls, and finally turbulent convection [161]. This sequence of states is similar to the concept of bifurcations in dynamical systems theory, offering an array of opportunities to investigate how order emerges from chaos in situations that are far from equilibrium.

The various manifestations of order arising from chaos in both natural and artificial systems are presented in Table 5. This overview demonstrates the prevalence of self-organisation phenomena across various scales, including microscopic chemical reactions, macroscopic geological processes, and complex biological and social systems. Table 5 demonstrates that the principles of self-organisation and the emergence of order from chaos extend beyond the Belousov-Zhabotinsky (BZ) reaction illustrated in Figs. 9 and 10. These phenomena are observed across various scales and disciplines. The Benard cell convection system illustrates spatial pattern formation similar to the BZ reaction, whereas slime mould aggregation illustrates chemical oscillations similar to those depicted in Fig. 10. The systems presented in Table 5 exhibit common themes, regardless of their visible differences. The emergence of coherent structures or behaviours from seemingly random initial conditions, the presence of oscillatory or wave-like phenomena, and the critical role of nonequilibrium conditions in sustaining these organised states are interesting phenomena. These shared characteristics highlight Prigogine’s claim that systems operating far from equilibrium have an essential ability for self-organisation [13]. Table 5 especially includes phenomena such as consciousness and the behaviour of social insects. The examples presented broaden the concept of emergent order to include complex biological and social systems, indicating that the principles identified in simpler chemical systems, such as the BZ reaction, might significantly improve our understanding of life’s organisation and cognitive processes [27]. The phenomena illustrated in Table 5 highlight the interdisciplinary aspects of research concerning self-organisation and emergent order. The principles of order arising from chaos are evident across various disciplines, including physics (laser emission), biology (Turing patterns), geology (sand dune formation), and cognitive science (consciousness) [27]. Comparing these diverse systems to the extensively researched BZ reaction (Figs. 9 and 10) allows researchers to derive insights into the fundamental principles that govern spontaneous pattern formation and self-organisation across various scientific domains. The comparative approach illustrated in Table 5 aligns with Prigogine’s perspective on developing a dialogue between humans and the natural world, acknowledging the creative and organising capabilities provided by nonequilibrium processes [115].

**Table 5:** Various forms of order arise from chaos across multiple scales and fields of study. This table demonstrates the wide distribution of self-organisation phenomena across various scales in nature, encompassing microscopic chemical reactions, macroscopic geological processes, and the emergence of consciousness. Each system illustrates fundamental principles of nonequilibrium thermodynamics and self-organisation, as investigated by Prigogine and exemplified by the Belousov-Zhabotinsky (BZ) reaction. The phenomena exhibit common themes, including pattern formation, oscillatory behaviour, and the emergence of coherent structures or behaviours from initially random or chaotic conditions. These systems collectively highlight Prigogine’s innovative viewpoint regarding the creative and organising capabilities of nonequilibrium processes in nature, challenging conventional interpretations of entropy and disorder. Drawing parallels between these diverse systems and the extensively studied BZ reaction (Figs. 1 and 2) provides insights into the universal principles that govern the spontaneous emergence of order across various scales and scientific domains

System	Self-organisation phenomenon	Relation to BZ reaction/Prigogine’s concepts
Benard cell convection	Hexagonal convection cells form in heated fluid	Spatial pattern formation, dissipative structures
Slime mould aggregation	Periodic cAMP pulses lead to spiral waves	Chemical oscillations, emergent patterns
Firefly synchronisation	Spontaneous synchronisation of light emissions	Coupled oscillators, collective behaviour
Turing patterns	Reaction–diffusion patterns in biological systems	Spatial pattern formation, morphogenesis
Sand dune formation	Regular patterns from wind-blown sand	Macroscopic self-organisation, emergent order
Laser emission	Transition from incoherent to coherent light	Phase transitions, bifurcations
Consciousness	Coordinated neural activity leading to awareness	Emergent complexity, oscillatory behaviour
Social insect colonies	Complex structures from simple individual rules	Collective intelligence, emergent order

**Fig. 11:**
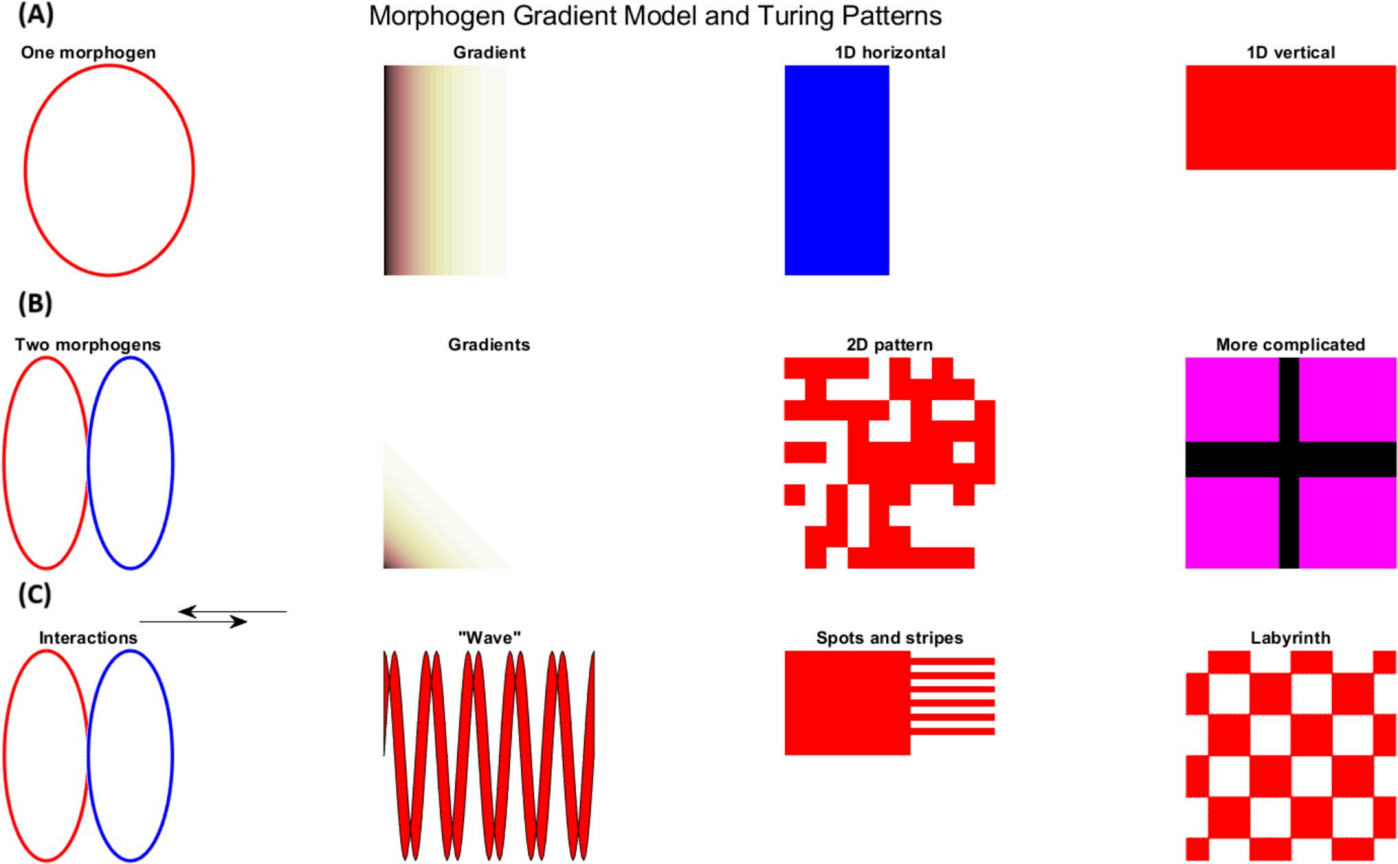
Schematic representation of morphogen gradient models and Turing patterns. **A** Single morphogen system: a gradient forms from a localised source, leading to 1D patterns. **B** Two-morphogen system: intersecting gradients create 2D patterns of increasing complexity. **C** Morphogen interactions: feedback loops between morphogens generate diverse spatial patterns. The progression from simple gradients to complex patterns illustrates the emergence of spatial organisation in biological systems, from basic positional information to intricate tissue structures. This figure demonstrates how increasing the number of morphogens and their interactions can lead to a wide range of pattern complexities, from simple stripes to labyrinthine structures, mirroring the diversity observed in natural systems

The emergence of spatial patterns in biological systems is elucidated through morphogen gradients and Turing mechanisms, as depicted in Fig. 11. This schematic illustrates the transition from simple, one-dimensional patterns to complex, two-dimensional structures resulting from the interaction of various morphogens. Figure 11A illustrates that a single morphogen can create a fundamental gradient, thereby offering positional information along a single axis. This principle is essential in developmental biology, elucidating phenomena such as anterior–posterior patterning in early embryos [173]. The resulting one-dimensional horizontal or vertical patterns resemble basic body plan organisations. The introduction of a second morphogen (Fig. 11B) significantly enhances the complexity of potential patterns. The convergence of two gradients facilitates the creation of two-dimensional positional information, thereby permitting more complex spatial arrangements. This concept is essential for understanding the development of complex organs and tissues, as cells have to determine their position in multiple dimensions [174]. Figure 11C demonstrates how interactions among morphogens can result in the emergence of self-organising Turing patterns. The patterns, initially proposed by Alan Turing [87], arise from the interaction between activator and inhibitor morphogens. The resulting structures—spots, stripes, and labyrinths—exhibit a notable similarity to patterns found in nature, including animal coat markings and vegetation arrangements in arid ecosystems [165].

The progression illustrated in Fig. 11 clarifies the mechanisms underlying biological pattern formation and exemplifies how simple rules can lead to complex outcomes. The principle of emergence, which describes how complex global patterns develop from local interactions, is fundamental to contemporary systems biology and reflects concepts present in Prigogine’s research on self-organisation in nonequilibrium systems [13].

## Conclusion

The study of spontaneous oscillations in proteinoid and kombucha systems offers compelling evidence for the emergence of order from chaos in prebiotic and early biological systems. Our research has demonstrated that these oscillations, which are the result of complex metabolic and chemical interactions, are essential for the preservation of dynamic equilibrium and self-organisation. A sophisticated level of coordination is demonstrated in kombucha by the symbiotic community of bacteria and yeast, which generates rhythmic fluctuations in biochemical components. This behaviour may be indicative of early ecosystem dynamics. In the same direction, the periodic structural and functional changes observed in proteinoids provide an insight into the potential contributions of primitive protein-like structures to the evolution of more complicated biological processes. The oscillatory behaviours observed in modern living organisms and the parallels between these systems suggest a continuity in the organisational principles of nature from prebiotic chemistry to contemporary biology. The fundamental significance of these oscillations in the transition from nonliving to living matter is made clear by their spontaneous nature, which could potentially represent a critical step in the origin of life. Additionally, our findings have substantial implications that extend beyond the realm of origin-of-life research. They provide novel approaches to the design of self-organising networks and adaptive systems, offering new perspectives for synthetic biology and biotechnology. The principles outlined in this study have the potential to inform the development of biomimetic materials and systems that are more adaptable and responsive. Future research should concentrate on the precise mechanisms that connect chemical fluctuations to the emergence of biological functions as we continue to resolve the complexities of these oscillatory systems. Furthermore, investigating the potential for these systems to demonstrate greater complexity behaviours, such as memory or learning, could offer additional insights into the basic principles of life.

## Data Availability

No datasets were generated or analysed during the current study.
